# Association Between Serum Testosterone Levels and Coronary Artery Stenosis: A Cross-Sectional Study in Central European Population

**DOI:** 10.3390/diagnostics16050814

**Published:** 2026-03-09

**Authors:** Pavol Fülöp, Zuzana Pella, Tibor Porubän, Peter Hreško, František Pavol Zajac, Mariana Dvorožňáková, Štefan Tóth, Dominik Pella

**Affiliations:** 1 2nd Department of Cardiology, Faculty of Medicine, East Slovak Institute of Cardiovascular Diseases, Pavol Jozef Šafárik University in Košice, Ondavská 8, 040 11 Košice, Slovakia; 2 Department of Cybernetics and Artificial Intelligence, Faculty of Electrical Engineering and Informatics, Technical University of Košice, 040 01 Košice, Slovakia; 3 1st Department of Cardiology, Faculty of Medicine, East Slovak Institute of Cardiovascular Diseases, Pavol Jozef Šafárik University in Košice, Ondavská 8, 040 11 Košice, Slovakia; 4 Department of Imaging Techniques, Faculty of Medicine, East Slovak Institute of Cardiovascular Diseases, Pavol Jozef Šafárik University in Košice, Ondavská 8, 040 11 Košice, Slovakia; 5 Department of Gerontology and Geriatrics, Faculty of Medicine, Pavol Jozef Šafárik University—University Hospital of St. Michael, Murgašova 1, 040 86 Košice, Slovakia

**Keywords:** testosterone, coronary artery disease, coronary stenosis, angiography, cardiovascular risk factors

## Abstract

**Background**: The relationship between testosterone and coronary artery disease (CAD) remains a subject of debate. Most studies suggest an inverse association—lower testosterone, higher risk. However, data from Central European populations undergoing coronary angiography are limited. **Objectives**: To investigate the association between serum testosterone levels and angiographically confirmed coronary artery stenosis in a Slovak population. **Methods**: This cross-sectional study included 129 consecutive stable patients (84 men, 45 women; mean age 64.3 ± 9.7 years) undergoing elective coronary angiography for suspected stable coronary artery disease. Significant coronary stenosis was defined as ≥50% luminal narrowing in any major epicardial vessel. Serum testosterone, lipid profile, and traditional risk factors were assessed. Univariate and multivariate logistic regression models were constructed to evaluate independent associations of coronary stenosis. **Results**: Coronary stenosis ≥ 50% was present in 74 patients (57.4%). Notably, patients with stenosis had significantly higher testosterone levels (6.62 ± 2.79 vs. 4.85 ± 3.50 ng/mL, *p* = 0.002). In univariate analysis, testosterone showed a significant association (OR 1.197 per ng/mL, OR 1.784 per SD, *p* = 0.003). In multivariate analysis adjusted for age, sex, diabetes mellitus, and LDL (low-density lipoprotein) cholesterol, testosterone remained independently associated (adjusted OR 2.043 per SD, 95% CI 1.221–3.420, *p* = 0.007), as did diabetes mellitus (OR 2.60, *p* = 0.032). **Conclusions**: Elevated serum testosterone is paradoxically associated with increased prevalence of coronary stenosis in our cohort. These findings from stable, chronic CAD patients may work fundamentally differently from what is observed in acute coronary syndromes, where stress-induced testosterone suppression may confound observed associations.

## 1. Introduction

Coronary artery disease (CAD) remains the world’s leading cause of mortality and morbidity worldwide, accounting for approximately 9 million deaths annually [1]. Traditional risk factors, including age, hypertension, diabetes mellitus, dyslipidemia, and smoking, have been well-established as predictors of CAD development and progression [2]. However, these factors do not fully explain the variability in CAD prevalence and severity, particularly the observed sex differences, suggesting that additional biomarkers may improve risk stratification [3].

Testosterone, the primary male sex hormone, has emerged as a potential modulator of cardiovascular health. Epidemiological studies have yielded conflicting results regarding the relationship between testosterone levels and CAD. Most studies report an inverse association—lower testosterone is linked to increased cardiovascular risk [4,5,6], worse outcomes [7,8], and more severe CAD [8,9]. This led researchers to hypothesize that testosterone might actually protect the heart. The proposed mechanisms include favorable effects on lipid metabolism [4], improved insulin sensitivity [5], better endothelial function [6], and dampened inflammatory pathways [10].

However, the existing literature has some important blind spots. First, most studies come from Western Europe, North America, or Asia [9,11,12,13]. Central and Eastern Europe are underrepresented. This matters because ethnic and racial differences in testosterone metabolism, androgen receptor (AR) polymorphisms, and cardiovascular disease patterns could modify the testosterone-CAD relationship [14,15]. Findings from one population cannot be directly extrapolated to others.

Second, many studies mixed in patients with acute coronary syndrome (ACS). During acute events, stress hormones suppress testosterone rapidly [16,17]. This creates a confounding problem—we cannot tell whether low testosterone caused the event or whether the event drove testosterone down. Third, sex-specific analyses often get short shrift, despite obvious differences in how testosterone works in men versus women [8,9]. Finally, the interaction between testosterone and other cardiovascular risk factors, particularly lipid profile and metabolic comorbidities, has been insufficiently explored [7,10].

Our study aimed to fill these gaps. We investigated the association between serum testosterone and angiographically confirmed coronary stenosis in a Central European (Slovak) population. Critically, we focused on stable patients undergoing elective coronary angiography. We hypothesized that testosterone would be independently associated with coronary stenosis after adjusting for traditional cardiovascular risk factors and lipid profile. Our comprehensive multivariate approach allowed us to determine testosterone’s association compared to established risk factors. We also explored whether comorbidities modify the effect.

## 2. Materials and Methods

### 2.1. Study Design and Population

The study population consisted of patients referred for coronary angiography (CAG) due to suspected atherosclerosis of coronary arteries, but without a history of previously documented coronary artery disease (either acute or chronic coronary syndromes). Patients were systematically selected from the 1st Department of Cardiology of East Slovak Institute for Cardiovascular Diseases (ESICD) based on the presence of one or more established cardiovascular risk factors and evidence of subclinical atherosclerosis detected by CT angiography or other non-invasive imaging modalities or exercise testing. All enrolled patients underwent selective coronary angiography (diagnostic and, when indicated, therapeutic intervention) along with comprehensive laboratory assessment.

Inclusion criteria: Adult patients (≥18 years) with one or more cardiovascular risk factors (hypertension, diabetes mellitus, dyslipidemia, smoking, family history of premature CAD) and evidence of subclinical atherosclerosis on non-invasive testing (CT angiography, stress echocardiography, or exercise electrocardiography) referred for elective coronary angiography.

Exclusion criteria: Known coronary artery disease (prior myocardial infarction, percutaneous coronary intervention, or coronary artery bypass grafting), acute coronary syndrome at presentation (ST-elevation myocardial infarction or non-ST-elevation acute coronary syndrome within 30 days), known testosterone replacement therapy, severe hepatic or renal dysfunction (creatinine > 200 μmol/L), active malignancy, or incomplete biochemical data.

### 2.2. Clinical and Biochemical Assessment

Clinical and biochemical data were collected following the standardized KSC MR Study protocols [18]. Detailed medical history was obtained, including cardiovascular risk factors, previous cardiovascular events, and current medications. Diabetes mellitus was defined as a previous diagnosis, use of antidiabetic medications, or fasting glucose ≥ 7.0 mmol/L. Hypertension was defined as a previous diagnosis, use of antihypertensive medications, or blood pressure ≥ 140/90 mmHg. Smoking status was categorized as current smoker, ex-smoker (quit > 6 months), or never smoker. History of ischemic heart disease included previous myocardial infarction, percutaneous coronary intervention, or coronary artery bypass grafting. Physical examination included measurement of height, weight, and blood pressure. Body mass index (BMI) was calculated as weight (kg) divided by height squared (m^2^).

Venous blood samples were collected after overnight fasting (≥12 h) prior to angiography. Serum was separated by centrifugation and stored at −80 °C until analysis. Biochemical parameters, including glucose, creatinine, total cholesterol, LDL cholesterol (low-density lipoprotein), HDL (high-density lipoprotein) cholesterol, and triglycerides, were measured using standard enzymatic methods on an automated analyzer. High-sensitivity C-reactive protein (CRP) was measured by immunoturbidimetric assay.

Testosterone was measured using a competitive enzyme-linked immunosorbent assay (ELISA; Abcam ab108666, Cambridge, UK). Manufacturer-specified reference ranges are 0.2–1.2 ng/mL for women and 1.8–9.0 ng/mL for men. Samples were collected in the morning (07:00–10:00) to minimize diurnal variation. The assay demonstrates sensitivity of 0.10 ng/mL with intra-assay and inter-assay coefficients of variation < 7.0% and <8.3%, respectively. Cross-reactivity with other steroids is minimal: dihydrotestosterone 2.03%, 17β-estradiol 0.16%, androstenedione 0.01%, Dehydroepiandrosteron (DHEA-S) 0.0%, and cortisol 0.01%. Given typical female dihydrotestosteron (DHT) concentrations (10–40 pg/mL), the contribution of DHT cross-reactivity to measured testosterone is negligible (<0.001 ng/mL). All samples were analyzed in duplicate, and measurements were within the assay detection range.

Menopausal status was assessed in all female participants through clinical history. Among the 45 women in our cohort, 2 (4.4%) were perimenopausal and 43 (95.6%) were postmenopausal. Perimenopausal status was defined as irregular menstrual cycles with vasomotor symptoms within the preceding 12 months. Postmenopausal status was defined as absence of menstruation for at least 12 consecutive months.

### 2.3. Coronary Angiography

Coronary angiography was performed via a radial or femoral approach using standard techniques. Multiple projections were obtained for each coronary artery. Coronary stenosis was assessed by two experienced interventional cardiologists blinded to clinical and biochemical data. The following coronary segments were evaluated: left main (LM), left anterior descending (LAD), diagonal branches, left circumflex (LCx), marginal branches, right coronary artery (RCA) and right interventricular posterior artery.

Primary outcome: Significant coronary stenosis was defined as ≥50% luminal diameter narrowing in any major epicardial vessel or major branch. This threshold was chosen based on established guidelines and functional significance [19].

### 2.4. Statistical Analysis

Continuous variables were tested for normality using the Shapiro-Wilk test. Normally distributed variables are presented as mean ± standard deviation (SD). Group comparisons were performed using Student’s *t*-test for continuous variables and chi-square test for categorical variables. Statistical significance was set at *p* < 0.05 (two-tailed).

Univariate logistic regression was performed to assess the individual association of each variable with coronary stenosis ≥ 50%. Multivariate logistic regression was conducted using a stepwise approach with progressive inclusion of variables. Variables for inclusion in the final model were selected a priori based on: (1) clinical relevance as established cardiovascular risk factors (age, sex, diabetes mellitus, LDL cholesterol), (2) univariate association with *p* < 0.10, (3) testosterone as the primary exposure of interest, and (4) maintaining adequate events-per-variable ratio (EPV ≥ 10) to minimize overfitting risk. The final model included 5 predictors (testosterone, age, sex, diabetes, LDL cholesterol), yielding EPV of 14.4 (72 events/5 predictors). HDL cholesterol, despite borderline univariate significance (*p* = 0.059), was excluded due to collinearity with LDL and EPV constraints. All continuous variables were standardized before inclusion to enable direct comparison of effect sizes. Results are presented as odds ratios (ORs) with 95% confidence intervals (CIs). Model performance was assessed using the area under the receiver operating characteristic curve (AUC).

Post hoc power calculation: For the primary univariate comparison, based on the observed difference in testosterone levels between groups with and without stenosis (mean difference 1.77 ng/mL, pooled SD 3.13 ng/mL, Cohen’s d = 0.565), our sample of 129 patients (72 with stenosis, 57 without) provides 89% statistical power at α = 0.05 (two-tailed). For multivariable logistic regression, our primary model includes 5 predictors with 72 events, yielding an events-per-variable (EPV) ratio of 14.4. This exceeds the minimum acceptable threshold of 10 but remains at the lower boundary for optimal stability. Sex-stratified multivariable analyses in women (*n* = 45, 20 events) achieve EPV of 3.3–5.0 (for 4–6 predictors)—substantially below the minimum threshold of 10. Consequently, sex-stratified multivariable results should be considered strictly exploratory and hypothesis-generating.

All statistical analyses were performed using Python version 3.12 with scipy.stats library version 1.11.0. Figures were generated using Python matplotlib version 3.7.1 and seaborn version 0.12.2 libraries. Claude Sonnet 4.5 (Anthropic) was used as a coding assistant to generate analysis scripts. Two-tailed *p*-values < 0.05 were considered statistically significant. No adjustment for multiple comparisons was applied, as all analyses tested pre-specified hypotheses based on biological plausibility.

## 3. Results

### 3.1. Baseline Characteristics

Our cohort consisted of 129 patients (mean age 64.3 ± 9.7 years). Men made up the majority at 65.1% (*n* = 84), with women making up 34.9% (*n* = 45). Significant coronary stenosis—defined as ≥50% luminal narrowing—was documented in just over half of participants (55.8%, *n* = 72). The remaining 44.2% (*n* = 57) showed no significant stenotic lesions. It is worth emphasizing that all patients were clinically stable at enrollment and underwent elective angiography.

When we compared baseline characteristics between patients with and without stenosis (Table 1), several patterns emerged. Patients who had stenosis demonstrated markedly elevated serum testosterone compared to those without (6.62 ± 2.79 versus 4.85 ± 3.50 ng/mL, *p* = 0.002), and this difference was statistically significant. We also noted that diabetes mellitus appeared to be more common in the stenosis group, though this only approached significance (36.1% versus 19.3%, *p* = 0.057). Other baseline variables—including age, sex distribution, BMI, presence of hypertension, prior history of ischemic heart disease, and current smoking status—showed no meaningful differences between the groups.

### 3.2. Univariate Variables of Coronary Stenosis

Table 2 shows our univariate logistic regression results. Testosterone had a significant association with coronary stenosis presence (OR 1.197 per ng/mL, 95% CI 1.064–1.346, *p* = 0.003). To put this in perspective, a one-standard-deviation increase in testosterone (SD = 3.22 ng/mL) translates to approximately 75% higher odds of having stenosis (OR 1.784 per SD, *p* = 0.003). Diabetes mellitus also predicted stenosis significantly (OR 2.364, 95% CI 1.046–5.339, *p* = 0.039). Female sex showed what might be interpreted as a protective trend (OR 0.721, 95% CI 0.607–0.857), though there was no statistical significance (*p* = 0.057). HDL cholesterol did not demonstrate statistical significance (OR 0.712, 95% CI 0.599–0.846, *p* = 0.059).

Odds ratios for continuous variables (age, BMI, testosterone, lipid parameters, CRP) are reported per unit increase as specified. For lipid parameters (cholesterol, LDL, HDL, triglycerides), OR represents the change per 0.1 mmol/L increase to provide clinically meaningful effect sizes. For testosterone, OR is reported both per ng/mL and per standard deviation (SD = 3.22 ng/mL) to facilitate interpretation. Statistical methods: Univariate logistic regression was performed for each variable separately. Odds ratios and 95% confidence intervals were calculated using maximum likelihood estimation, and *p*-values were derived from likelihood ratio tests. All tests were two-sided. Abbreviations: OR = odds ratio; CI = confidence interval; BMI = body mass index; CAD = coronary artery disease; LDL = low-density lipoprotein; HDL = high-density lipoprotein; CRP = C-reactive protein; ESC = European Society of Cardiology.

To probe deeper into how testosterone relates to disease severity, we stratified our cohort by degree of stenosis. The relationship showed what appeared to be a dose-response pattern (Table 3). Patients without stenosis had testosterone levels of 4.25 ± 3.55 ng/mL. Those with moderate stenosis (50–69%) showed borderline significantly higher levels at 6.12 ± 2.68 ng/mL (*p* = 0.050). In patients with severe stenosis (≥70%), testosterone levels reached 6.81 ± 2.84 ng/mL (*p* < 0.001), representing a 60% increase over the no-stenosis group. ANOVA confirmed overall group differences (*p* = 0.0045), and linear trend analysis showed progressive increases (r = 0.359, *p* < 0.0001).

### 3.3. Multivariate Analysis: Progressive Models

To ensure adequate statistical power and minimize overfitting risk, we constructed our primary multivariate model with five predictors, achieving an events–per-variable ratio of 14.4 (72 events/5 predictors). This exceeds the minimum acceptable threshold of 10 and approaches the preferred threshold of 15. The model included testosterone (primary predictor of interest), age, sex, diabetes mellitus, and LDL cholesterol—variables selected based on clinical relevance and strength of univariate associations.

Table 4 presents the results of our primary multivariate model with standardized predictors. Testosterone maintained its association with coronary stenosis (adjusted OR 2.043 per standard deviation, 95% CI 1.221–3.420, *p* = 0.007). Given a testosterone standard deviation of 3.22 ng/mL, this corresponds to approximately 95% higher odds of stenosis for a patient with testosterone 9.06 ng/mL versus 5.84 ng/mL (population mean), after adjusting for all other variables in the model.

Diabetes mellitus also showed a strong association (OR 2.600, 95% CI 1.085–6.229, *p* < 0.032). However, associations with LDL cholesterol (OR 1.467 per SD, 95% CI 0.985–2.186, *p* = 0.060) and age (OR 1.342 per SD, 95% CI 0.907–1.987, *p* = 0.141) were non-significant. Female sex showed a non-significant lower odds of stenosis (OR 0.892, 95% CI 0.708–1.123, *p* = 0.330).

The apparent model performance (evaluated on the same data used for fitting) yielded an AUC of 0.716 (95% CI 0.633–0.799). However, apparent performance typically overestimates true performance, particularly with smaller sample sizes. To obtain more realistic performance estimates, we conducted rigorous internal validation.

Bootstrap optimism correction using 500 resamples revealed a mean optimism of 0.036 (SD 0.042). This indicates the apparent AUC overestimates true performance by approximately 3.6 percentage points. The optimism-corrected AUC was 0.680, representing a more realistic estimate of model discrimination. An optimism of 0.036 is considered moderate and acceptable for our sample size.

Ten-fold cross-validation, which evaluates model performance on data not used for fitting, yielded a mean AUC of 0.655 (SD 0.108, range 0.475–0.833). The 95% confidence interval for cross-validated performance was (0.442–0.867). The difference between apparent and cross-validated AUC (0.061) is consistent with moderate optimism and indicates acceptable model stability. The consistency between bootstrap-corrected AUC (0.680) and cross-validated AUC (0.655) provides confidence in our validation approach and confirms moderate discrimination capacity with acceptable stability.

### 3.4. Sex-Stratified Analysis

To investigate potential sex-specific differences in the testosterone-stenosis relationship, we performed stratified analyses for men and women separately. Using the manufacturer-specified reference range (0.2–1.2 ng/mL), elevated total testosterone (>1.2 ng/mL) was observed in 28 women (62.2%). However, this should be interpreted with caution given the absence of sex hormone-binding globulin (SHBG) measurement and the well-documented limitations of ELISA methodology at low female concentrations. Without free-testosterone assessment, we cannot determine whether these values represent true androgen excess or reflect elevated SHBG, assay variability, or other confounding factors.

**Figure 1:**
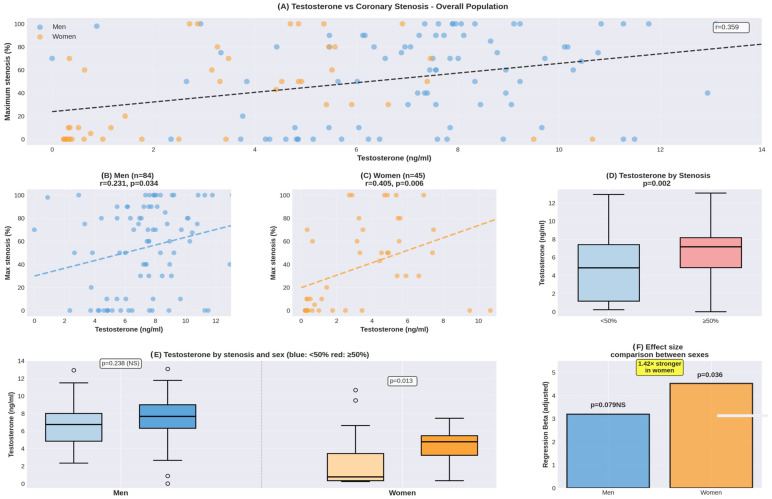
Testosterone levels and coronary stenosis. (**A**) Scatter plot showing the relationship between serum testosterone levels (x-axis, ng/mL) and maximum coronary stenosis percentage (y-axis, %) for the overall population (N = 129). The linear regression line (black dashed) with a 95% confidence interval (shaded gray region) shows a significant positive correlation (r = 0.359, *p* < 0.001). (**B**) Scatter plot for men only (*n* = 84). (**C**) Scatter plot for women only (*n* = 45). (**D**) Box plots comparing testosterone levels between patients with <50% stenosis (light blue box, *n* = 57) and ≥50% stenosis (pink/red box, *n* = 72). (**E**) Box plots stratified by both stenosis severity and sex. The left panel shows men: a blue box for <50% stenosis and a darker blue box for ≥50% stenosis. The right panel shows women: a light orange box for <50% stenosis and a darker orange box for ≥50% stenosis. (**F**) Bar chart showing regression beta coefficients (effect sizes) comparing the strength of the testosterone-stenosis association between sexes. The blue bar represents men and the orange bar represents women. Abbreviations: r = Pearson correlation coefficient; NS = not significant; Beta = standardized regression coefficient (effect size).

In crude correlation analyses, testosterone showed positive associations with maximum stenosis in both sexes (Figure 1). However, the correlation in women (r = 0.405, *p* = 0.006) was notably more robust than that in men (r = 0.231, *p* = 0.034). When exploring these associations with multivariable adjustment for age, BMI, and traditional risk factors, women showed a numerically stronger association (adjusted β = 4.525, *p* = 0.036, R^2^ = 0.254) compared to men (adjusted β = 3.195, *p* = 0.079, R^2^ = 0.090). However, given the small female sample size (*n* = 45, 20 events) and critically low events-per-variable ratio (EPV = 3.3–5.0 for 4–6 predictors), these multivariable-adjusted estimates are statistically unstable and unreliable. The wide confidence intervals and borderline *p*-values reflect this instability rather than robust associations. These sex-stratified multivariable results are presented as strictly exploratory and hypothesis-generating only, requiring validation in adequately powered cohorts (minimum 150–200 women) before drawing firm conclusions.

When we looked at the dichotomous outcome (stenosis ≥ 50% vs. <50%), the sex difference became even more apparent. Women with significant stenosis had markedly higher testosterone than those without. Given the non-normal distribution in the no-stenosis group (Shapiro-Wilk *p* < 0.001, skewness = 1.51), we report both parametric and non-parametric statistics: mean ± SD (4.37 ± 1.93 vs. 2.35 ± 3.04 ng/mL) and median (IQR) [4.78 (3.23–5.47) vs. 0.76 (0.33–3.43) ng/mL]. The Mann-Whitney U test confirmed a highly significant difference (U = 380, *p* = 0.003). The effect size was substantial (Cohen’s d = 0.773; rank-biserial correlation = −0.52). In comparison, men showed a much smaller effect (Cohen’s d = 0.269, *p* = 0.238). The adjusted beta coefficient for testosterone was 1.42-fold higher in women, indicating that the testosterone-stenosis link operates quite differently—and more strongly—in the female cohort.

**Figure 2:**
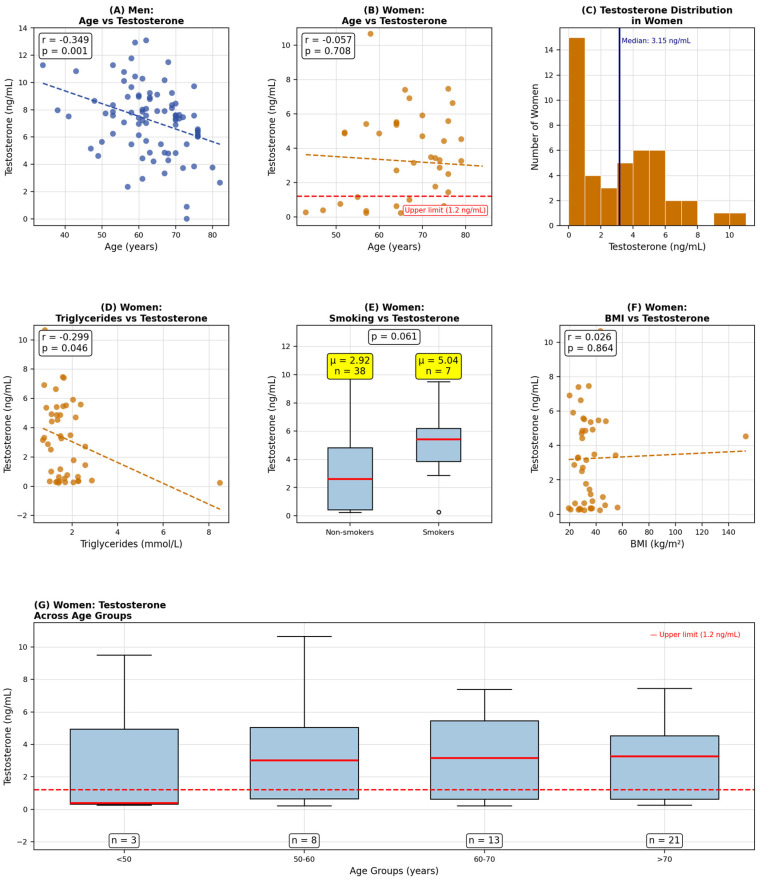
Association between age, sex, and serum testosterone levels. (**A**) Scatter plot showing the relationship between age (x-axis, years) and serum testosterone levels (y-axis, ng/mL) in men (*n* = 84, blue circles). (**B**) Scatter plot showing the relationship between age (x-axis, years) and serum testosterone levels (y-axis, ng/mL) in women (*n* = 45). (**C**) Histogram showing the distribution of testosterone levels in women (*n* = 45). The vertical axis shows the number of women, and the horizontal axis shows testosterone levels (ng/mL). The median testosterone level (3.15 ng/mL) is marked by a vertical blue line. (**D**) Scatter plot displaying the relationship between serum triglycerides (TG, x-axis, mmol/L) and testosterone levels (y-axis, ng/mL) in women (*n* = 45). (**E**) Box plots comparing testosterone levels between non-;smokers (*n* = 38) and smokers (*n* = 7) in women. (**F**) Scatter plot showing the relationship between body mass index (BMI, x-axis, kg/m^2^) and testosterone levels (y-axis, ng/mL) in women (*n* = 45). (**G**) Box plots comparing testosterone levels across four age groups in women: <50 years, 50–60 years, 60–70 years, and >70 years. Abbreviations: BMI = body mass index; r = Pearson correlation coefficient.

Supplementary analyses in women revealed that total testosterone levels were not significantly associated with BMI (Pearson r = −0.088, *p* = 0.570) or metabolic syndrome components (Figure 2). The prevalence of elevated total testosterone was similar across BMI categories (*p* = 0.926) and metabolic syndrome status (*p* = 0.161). However, this absence of expected correlations raises concerns about confounding by unmeasured SHBG, which typically increases with lower BMI and could artifactually elevate total testosterone measurements in leaner women while free testosterone remains normal. Without SHBG data, we cannot distinguish true androgen excess from binding protein variation.

### 3.5. Multi-Vessel Disease Analysis

Multi-vessel disease was present in 69 patients (53.5%): 43 patients (33.3%) had one-vessel disease, 16 patients (12.4%) had two-vessel disease, and 13 patients (10.1%) had three-vessel disease (Table 5). Sex differences in disease extent were apparent—61.9% of men (*n* = 52) had multi-vessel disease compared to only 37.8% of women (*n* = 17), *p* = 0.008. Given the small sample size (20 events) and critically low events-per-variable ratio (EPV = 5.0), multivariable analysis was not performed in the women subgroup.

**Figure 3:**
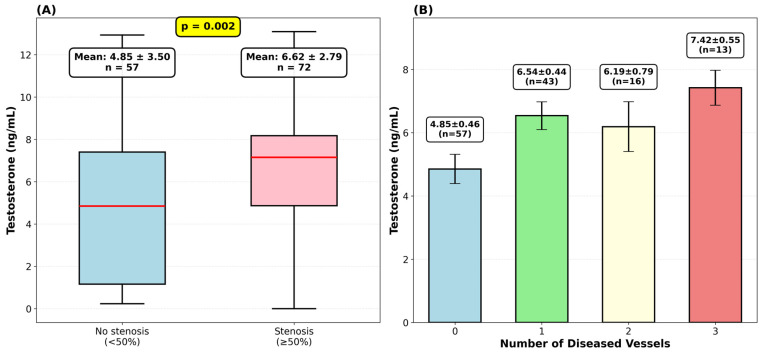
Testosterone and coronary artery disease. (**A**) Box plots showing testosterone levels in patients without stenosis (*n* = 57) versus those with stenosis ≥ 50% (*n* = 72). (**B**) Bar chart (mean ± standard error of the mean) demonstrating progressive increase in testosterone levels with extent of coronary disease.

We wanted to see whether our testosterone findings held up using an alternative measure of disease severity—the number of diseased vessels rather than maximum stenosis percentage. A vessel was classified as diseased if any segment showed ≥50% stenosis. We analyzed the three main epicardial territories: first, the left anterior descending (LAD) system, which includes RIA, RD1, and RD2; second, the left circumflex (LCX) system, encompassing RCX, RMS1, RMS2, and RIM; and third, the right coronary artery (RCA) system, consisting of ACD and RIP. The extent of coronary artery disease showed a positive correlation with testosterone levels (Pearson r = 0.253, *p* = 0.004). Mean testosterone levels progressively increased from 4.85 ± 3.50 ng/mL in patients without significant disease to 7.42 ± 1.99 ng/mL in those with three-vessel disease, suggesting a dose-response relationship between testosterone and disease burden (Figure 3).

## 4. Discussion

### 4.1. Principal Findings

Our cross-sectional study of 129 patients undergoing coronary angiography yielded findings that run counter to much of the prevailing wisdom about testosterone and cardiovascular health. First, we found a positive association between serum testosterone and angiographically confirmed stenosis. Patients with stenosis had significantly higher testosterone levels—6.62 versus 4.85 ng/mL (*p* = 0.002). This was not what we expected based on the epidemiological literature.

Second, in multivariate analysis adjusted for age, sex, diabetes mellitus, and LDL cholesterol, testosterone remained strongly and independently associated with coronary stenosis (adjusted OR 2.043 per standard deviation, 95% CI 1.221–3.420, *p* = 0.007). Diabetes mellitus demonstrated a similar strength of association (OR 2.600, 95% CI 1.085–6.229, *p* = 0.032). Notably, traditional cardiovascular risk factors, including age and LDL cholesterol, did not show independent associations in our model (*p* = 0.141 and *p* = 0.060, respectively), possibly reflecting the specific characteristics of our cohort (stable, symptomatic patients with statin use).

Third, these associations proved stable under rigorous internal validation. Bootstrap analysis (500 resamples) revealed moderate optimism (0.036), yielding an optimism-corrected AUC of 0.680. Ten-fold cross-validation confirmed moderate discrimination (mean AUC 0.655, SD 0.108), with performance consistent across validation folds. The concordance between bootstrap and cross-validation results strengthens confidence that our findings are not artifacts of overfitting.

Our findings demonstrate remarkable consistency when quantifying coronary disease severity. When assessed by maximum stenosis percentage (a continuous measure of disease severity), testosterone showed strong positive correlations in both sexes, though notably stronger in women (r = 0.405, *p* = 0.006) compared to men (r = 0.231, *p* = 0.034).

It is critical to emphasize that our cross-sectional design cannot establish causality. The observed positive association between testosterone and stenosis may reflect: (1) testosterone contributing to atherosclerosis development, (2) reverse causality with chronic ischemia triggering compensatory testosterone elevation, (3) residual confounding by unmeasured factors associated with both testosterone and stenosis, or (4) complex bidirectional relationships. Our findings should be interpreted as hypothesis-generating signals requiring validation in longitudinal cohorts with repeated hormonal measurements and comprehensive metabolic phenotyping.

### 4.2. Testosterone Superiority over Traditional Risk Assessment

Our testosterone findings complement recent multi-biomarker work from our institution. Hostačná et al. [20] reported that growth differentiation factor 15 (GDF-15) demonstrated sex-specific patterns similar to testosterone. In their cohort, GDF-15 expression correlated with stenosis severity in men but not in women. This suggests that sex hormones and inflammatory cytokines may interact in complex, sex-specific ways to modulate atherosclerotic risk. Intriguingly, they also found a negative correlation between interleukin-6 and stenosis severity, which contradicts traditional inflammatory hypotheses of CAD. This mirrors our paradoxical testosterone results. Both findings challenge simplistic pro-inflammatory models of CAD pathogenesis. When independent biomarker panels from separate studies converge on similar conclusions about fundamental sex differences in CAD biology—differences that transcend individual molecular pathways—it strengthens the case that something important is going on here.

Of note, the ESC 10-year cardiovascular risk score [21]—designed for risk stratification in asymptomatic primary prevention populations—showed limited discrimination in our symptomatic angiography cohort (AUC 0.380). This finding illustrates a well-recognized methodological issue: primary prevention risk scores, which estimate future event probability using factors such as age, sex, smoking, blood pressure, and cholesterol, lose discriminative ability when applied to symptomatic patients already selected for high-risk clinical presentation. This represents spectrum bias [22], where selection of a high-risk symptomatic population (all patients referred for angiography) fundamentally alters test performance characteristics. Rather than representing a failure of the ESC score, this demonstrates the limitations of applying population-based risk prediction tools to disease detection in selected clinical cohorts.

The modest performance of CRP and LDL cholesterol, despite their established roles in atherosclerosis, deserves comment. CRP, while elevated in inflammatory states, demonstrates substantial day-to-day variability and lacks specificity for coronary disease. LDL cholesterol, though causally linked to atherosclerosis development, may show limited cross-sectional discrimination once disease is established, particularly in treated populations. In our cohort, 67.4% of patients were receiving statin therapy, potentially attenuating LDL’s discriminative capacity.

These findings suggest that testosterone is associated with coronary stenosis independently of traditional cardiovascular risk factors. Whether testosterone could contribute to cardiovascular risk assessment requires prospective validation studies with clinical outcome data.

Our findings stand in contrast to the prevailing view in testosterone-CAD research (see Table 6 for comparison). Most large epidemiological studies have reported an inverse relationship—lower testosterone, higher cardiovascular risk. The European Male Ageing Study (EMAS) found that low testosterone predicted incident cardiovascular events in middle-aged and elderly men [11]. Corona et al.’s meta-analysis of interventional studies demonstrated that testosterone and cardiovascular risk share a complex, dose-dependent relationship [12]. The Framingham Heart Study reported that lower testosterone correlated with greater vascular calcification burden [13]. These findings solidified the widely accepted hypothesis that testosterone is cardioprotective [10].

But there is a smaller, growing body of evidence that aligns with our paradoxical finding that higher testosterone is associated with increased stenosis. Several factors deserve consideration here—particularly differences in population characteristics, clinical presentation, comorbidity patterns, and ethnic/racial composition. The contrasting outcomes across studies likely reflect variations in study design (observational versus interventional), population characteristics (acute versus stable CAD, age ranges, comorbidity burden), testosterone measurement approaches (endogenous levels versus exogenous replacement), and clinical context.

### 4.3. Role of Acute Coronary Syndrome and Clinical Presentation

Our study diverges sharply from much of the published literature—we focused exclusively on stable patients. Acute myocardial ischemia unleashes profound neuroendocrine stress responses. One of these is suppression of the hypothalamic-pituitary-gonadal axis, which causes testosterone to plummet rapidly [16]. Pesonen et al. showed that testosterone levels can drop by 20–40% during acute coronary events and remain suppressed for weeks afterward [17].

In our cohort were only stable patients undergoing elective angiography. This is a fundamental difference from many testosterone-CAD studies that included substantial proportions of acute patients [16,17]. When ACS patients are included, the observed inverse association (low testosterone = more severe CAD) may reflect reverse causality—the acute event suppresses testosterone, rather than low testosterone causing the event.

What our findings suggest is that in stable, chronic CAD states—removed from the hormonal chaos of acute stress—the testosterone-stenosis relationship works differently. Trumble et al. reported something similar in a low-risk population, finding higher testosterone in stable CAD patients compared to controls [24,25]. This points to the possibility that the testosterone-CAD relationship might be bidirectional or time-dependent. Acutely suppressed during events, but chronically elevated when established atherosclerosis is quietly sitting there.

### 4.4. Sex-Specific Differences in the Testosterone-Stenosis Association

Our data suggest stronger testosterone-stenosis associations in women than in men, though the small female sample (*n* = 45, 20 events) limits definitive conclusions. Women with stenosis had markedly higher testosterone (median 4.78 vs. 0.76 ng/mL, Mann-Whitney *p* = 0.003), whereas men showed modest differences. However, critical measurement limitations constrain interpretation.

Without SHBG assessment, we cannot distinguish true androgen excess from elevated binding proteins. The 62% prevalence of elevated total testosterone (>1.2 ng/mL) in our female cohort, combined with absent BMI correlation, raises concerns about SHBG confounding or ELISA imprecision at low female concentrations [26]. SHBG typically increases with age and lower adiposity in postmenopausal women, artifactually elevating total testosterone while free testosterone remains normal.

In postmenopausal women, elevated androgens commonly accompany metabolic syndrome [27]. Hyperinsulinemia directly stimulates ovarian theca cells to increase testosterone production, synergistic with luteinizing hormone (LH) but without triggering hypothalamic-pituitary-gonadal (HPG) suppression as in men. This explains paradoxically higher testosterone in metabolically unhealthy postmenopausal women. Whether our findings reflect true androgen excess, SHBG variation, or methodological artifact cannot be determined without comprehensive hormonal profiling.

The observed sex differences in crude associations (women r = 0.405 vs. men r = 0.231) warrant further investigation but require validation in adequately powered cohorts (minimum 150–200 per sex) with free testosterone and SHBG measurement. Multivariable-adjusted sex-stratified results (EPV = 3.3–5.0 in women) are statistically unstable and presented as strictly exploratory.

### 4.5. Factors Contributing to Observed Associations

Several factors may contribute to the unexpected positive testosterone-stenosis association observed in our cohort, though we emphasize that these remain speculative hypotheses rather than established mechanisms.

First, our cohort’s high comorbidity burden (28.7% diabetes, 68.2% hypertension, mean BMI 31.7 kg/m^2^) may fundamentally alter testosterone’s vascular effects. Diabetes showed the strongest association with stenosis (OR 2.364), and the testosterone-stenosis link appeared to be stronger in diabetic versus non-diabetic patients. In metabolically healthy populations, testosterone generally shows protective cardiovascular effects [15], whereas our findings emerge from a population with established metabolic dysfunction. Whether testosterone operates differently in diseased versus healthy metabolic states remains unclear.

The direction of the obesity-testosterone relationship differs fundamentally between sexes. In men, excess visceral fat increases aromatase activity, converting testosterone to estradiol and suppressing the HPG axis—the well-described obesity-induced hypogonadism pathway [28,29]. In women, particularly postmenopausal, the picture is reversed. After menopause, declining estrogen production shifts the hormonal balance toward relative androgen predominance, while ovarian stromal theca cells continue testosterone synthesis under LH stimulation [30].

Obesity and insulin resistance amplify this process: hyperinsulinemia directly stimulates theca cell androgen production synergistically with LH, without triggering HPG suppression as seen in men. Concurrently, reduced SHBG increases the free-androgen fraction, and adipose tissue itself contributes through peripheral conversion of adrenal precursors to testosterone [31]. In postmenopausal women with metabolic syndrome—the predominant profile of our female cohort—obesity therefore promotes rather than suppresses hyperandrogenism. This sex-specific divergence likely contributes to the stronger testosterone-stenosis association observed in our female patients.

Second, ethnic variation may modify testosterone-CAD relationships. Our Central European (Slovak, predominantly Caucasian) population has been underrepresented in testosterone research, which has focused on Western European, North American, and Asian populations [13,32]. AR polymorphisms vary across ethnic groups and could theoretically influence tissue sensitivity to androgens [14]. However, whether ethnic differences contribute to our findings cannot be determined from our single-population study.

### 4.6. Correlation vs. Causation: Interpreting the Testosterone-Stenosis Association

We need to be clear-eyed about what our study can and cannot tell us. Our cross-sectional design shows a statistical link between elevated testosterone and stenosis severity, but it cannot establish which came first or whether one causes the other. Several alternative explanations deserve consideration.

First, testosterone elevation might be a response rather than a driver. Chronic myocardial ischemia triggers complex neuroendocrine shifts. Testosterone upregulation could be adaptive, similar to how neurohormones activate in heart failure [15]. This “reactive elevation” idea fits with our finding that the association is stronger in stable chronic CAD patients (our cohort) compared to acute presentations where stress–induced suppression dominates.

Second, testosterone might just be a marker of other unmeasured factors rather than an independent risk factor. Higher testosterone in women, for instance, is strongly associated with polycystic ovary syndrome (PCOS), metabolic syndrome, and insulin resistance [33,34]—all established CAD risk factors. While we adjusted for diabetes, BMI, and lipids, we cannot rule out residual confounding from inflammatory pathways, genetic factors, or AR variants [35,36].

Third, reverse causation is possible. Coronary atherosclerosis develops over decades and could influence hormonal regulation through systemic inflammation, oxidative stress, or altered liver metabolism. We need longitudinal studies with repeated testosterone measurements to untangle temporal relationships.

Our study sheds light on endogenous testosterone-CAD relationships in stable disease. But clinical decisions about testosterone replacement therapy (TRT) should follow the randomized trial evidence showing it is safe when prescribed appropriately.

### 4.7. Clinical Implications

Although our study examines endogenous testosterone and does not directly address TRT, the clinical implications for testosterone measurement and treatment deserve discussion.

Short–term TRT studies have shown metabolic benefits—improved insulin sensitivity, reduced visceral fat, and better endothelial function. Long–term cardiovascular safety was established by the TRAVERSE trial [23,37], a randomized, double–blind study of 5204 hypogonadal men, which demonstrated non–inferiority of TRT versus placebo for major cardiovascular events over a median of 3.1 years. This provides reassuring evidence for TRT in appropriately selected elderly men with symptomatic hypogonadism—a population with high prevalence of metabolic syndrome and CAD risk that closely mirrors patients undergoing coronary angiography in clinical practice.

Whether TRT directly modifies atherosclerotic burden remains an open question. TRAVERSE did not include serial coronary imaging, and while TRT improves several atherosclerosis-related intermediate endpoints, translation to measurable plaque regression has not been demonstrated. Our finding that higher endogenous testosterone is associated with greater stenosis severity may appear paradoxical in this context. However, endogenous testosterone elevation within established metabolic disease likely reflects compensatory or pathological processes fundamentally different from therapeutic normalization of deficiency. These scenarios should not be conflated when making clinical decisions about TRT.

Our findings should not discourage appropriate TRT in hypogonadal men. However, they suggest that testosterone measurement may be relevant for cardiovascular risk stratification in stable CAD populations, particularly in Central/Eastern European patients and women, where the hormonal context differs from that of well-studied Western cohorts.

### 4.8. Strengths and Limitations

Our study has notable strengths. We used gold-standard coronary angiography rather than surrogate markers. This is superior to non-invasive surrogates and enabled precise characterization across multiple stenosis categories (none, mild, moderate, severe) and vessel territories. However, slow coronary flow was not assessed. We have applied comprehensive multivariate modeling, included only stable patients to minimize acute stress-related hormonal confounding, collected systematic biochemical data, and studied an underrepresented Central European population with high comorbidity burden—reflecting real-world practice rather than pristine research cohorts.

However, important limitations require careful interpretation. First, our cross-sectional design represents a fundamental limitation. We demonstrate a statistical association between elevated testosterone and stenosis severity, but cannot establish temporal precedence or causality. Prospective longitudinal cohorts measuring baseline testosterone in stenosis-free patients and tracking development over years, or randomized trials measuring stenosis progression by serial angiography/CT, would strengthen causal inference—though ethical and practical constraints are formidable.

Second, our sample size (129 patients, 72 events) limits statistical power for subgroup analyses. The events-per-variable ratio of 14.4 (72 events/5 predictors) exceeds the minimum threshold of 10 but remains below the preferred 20, necessitating cautious interpretation.

Third, we measured only total testosterone without SHBG, preventing calculation of free or bioavailable testosterone. Since total testosterone is >99% protein-bound and only free testosterone is biologically active, and SHBG varies with age, obesity, insulin resistance, and thyroid function, our measurements cannot distinguish true androgen excess from altered binding. This limitation is particularly problematic for women. The observed 62.2% prevalence of elevated total testosterone (>1.2 ng/mL), combined with the unexpected absence of BMI correlation, raises serious concerns about SHBG confounding. In postmenopausal women, SHBG typically increases with age and lower adiposity, which would artifactually elevate total testosterone while free testosterone remains normal or low.

Fourth, we used ELISA without LC-MS/MS confirmation. ELISA has well-documented limitations at low female concentrations, including potential matrix effects and reduced accuracy. Systematic overestimation cannot be excluded.

Fifth, we lack extended androgen profiling (DHEA-S, androstenedione, 17-OH progesterone, estradiol, LH/FSH (follicle-stimulating hormone)) and clinical phenotyping (menstrual history, Ferriman-Gallwey scoring, ovarian imaging). These gaps prevent us from determining whether elevated testosterone represents PCOS (and which phenotype), adrenal disorders, or other etiologies.

Sixth, other limitations include single-center design (limiting generalizability), residual confounding from unmeasured variables (diet, physical activity, genetic polymorphisms, inflammatory cytokines), binary stenosis classification potentially not capturing the full atherosclerotic burden, selection bias inherent to angiography referral, and lack of systematic analysis of medication effects or previous anabolic steroid use.

Seventh, selective angiography has its own limitations. It visualizes luminal narrowing but does not show plaque composition, vulnerability, or functional significance. Testosterone might be associated with stable fibrous plaques rather than rupture-prone lesions, which our design cannot distinguish. Intravascular imaging or functional testing (fractional flow reserve) would provide complementary data. Also, angiography captures disease at a single time point. It cannot tell us when stenosis developed or whether testosterone elevation preceded, accompanied, or followed plaque progression. While we demonstrate correlation, proving that elevated testosterone causes plaque formation would require a different methodology.

We cannot distinguish whether elevated testosterone represents (1) a causal contributor to atherosclerosis, (2) a compensatory response to existing disease, (3) a marker of unmeasured pathological processes, or (4) a confounded association. Mechanistic speculation without prospective data, comprehensive hormonal profiling, and functional studies risks misleading narratives. The appropriate interpretation is as an unexpected signal warranting further investigation rather than evidence for specific mechanisms.

Finally, while angiography objectively quantifies stenosis and enables dose-response analyses across multiple categories and vessel territories, it visualizes luminal narrowing but not plaque composition, vulnerability, or functional significance. Testosterone might be associated with stable fibrous plaques rather than rupture-prone lesions, which our design cannot distinguish. Intravascular imaging or functional testing would provide complementary data. Angiography captures disease at a single time point and cannot establish whether testosterone elevation preceded, accompanied, or followed plaque progression. The appropriate interpretation of our findings is as an unexpected signal warranting further investigation rather than evidence for specific mechanisms.

### 4.9. Future Directions

Several research priorities emerge from our work. First, prospective longitudinal studies with larger samples (*n* ≥ 300) and extended follow-up are needed to examine testosterone-CAD relationships with adequate power for sex-stratified and comorbidity-specific analyses.

Second, multi-ethnic comparative studies are required to clarify whether our findings reflect ethnic-specific biology or apply more broadly.

Third, comprehensive hormonal profiling—free testosterone, SHBG, estradiol, DHT, LH, FSH—is necessary to identify which measures best predict CAD.

Fourth, comorbidity interaction studies examining how diabetes, obesity, and metabolic syndrome modify testosterone–CAD relationships are warranted.

Fifth, mechanistic investigations should be performed. What is AR expression like in coronary plaques? How does testosterone affect endothelial function? Which inflammatory pathways get activated?

Sixth, genetic association studies examining AR polymorphisms, aromatase variants, and testosterone metabolism genes in relation to CAD are required.

Until such data emerge, our findings should be interpreted as hypothesis-generating associations requiring validation in independent cohorts and mechanistic investigation in experimental models. The consistency of our findings across multiple analytical approaches—univariate, multivariate, dose-response, and multi-vessel disease—and the strong effect sizes strengthen biological plausibility. But causality remains unproven.

## 5. Conclusions

In this cross-sectional study of stable patients undergoing coronary angiography, we observed an unexpected positive association between serum testosterone and coronary stenosis severity—contrary to prevailing hypotheses of testosterone as cardioprotective. This “paradoxical” finding likely reflects the complexity of testosterone-cardiovascular relationships rather than a simple causal mechanism. Several factors may contribute, including our cohort’s stable clinical presentation (eliminating acute stress-induced suppression), high metabolic comorbidity burden, Central European ethnicity, and fundamental measurement limitations (absence of SHBG, free testosterone, and comprehensive hormonal profiling).

Critically, our cross-sectional design cannot establish causality or temporal relationships. The observed associations may reflect testosterone contributing to atherosclerosis, reverse causality with disease triggering compensatory hormonal responses, residual confounding by unmeasured metabolic and inflammatory factors, or complex bidirectional interactions. Our findings should be interpreted as hypothesis-generating signals indicating that testosterone-CAD relationships are more heterogeneous and context-dependent than previously recognized.

These results should not be extrapolated to testosterone replacement therapy decisions, where randomized trial evidence (TRAVERSE) supports cardiovascular safety in appropriately selected hypogonadal men. Rather, our findings underscore the need for prospective longitudinal research with: (1) repeated testosterone and SHBG measurements to establish temporal relationships, (2) comprehensive hormonal profiling including free testosterone, estradiol, and DHEA-S, (3) genetic assessment of AR variants, (4) detailed metabolic and inflammatory phenotyping, and (5) serial coronary imaging to assess plaque progression. Only such studies can definitively determine whether endogenous testosterone elevation in stable high-risk populations represents cause, consequence, or confounded association with coronary atherosclerosis.

## Figures and Tables

**Table 1:** Baseline characteristics of the study population stratified by coronary stenosis status.

Parameter	Stenosis ≥ 50% (*n* = 72)	Stenosis < 50% (*n* = 57)	*p*-Value
Age (years)	64.6 ± 9.4	63.9 ± 10.2	0.690
Male sex, *n* (%)	52 (61.9%)	32 (38.1%)	0.086
BMI (kg/m^2^)	31.2 ± 5.1	32.4 ± 7.7	0.273
Diabetes mellitus, *n* (%)	26 (36.1%)	11 (19.3%)	0.057
Hypertension, *n* (%)	46 (63.9%)	42 (73.7%)	0.319
Current smoker, *n* (%)	17 (23.6%)	9 (15.8%)	0.380
History of CAD, *n* (%)	19 (26.4%)	12 (21.1%)	0.415
Testosterone (ng/mL)	6.62 ± 2.79	4.85 ± 3.50	0.002
LDL cholesterol (mmol/L)	3.04 ± 1.10	2.87 ± 0.90	0.359
HDL cholesterol (mmol/L)	1.19 ± 0.27	1.28 ± 0.28	0.054
Triglycerides (mmol/L)	1.69 ± 0.78	1.66 ± 1.20	0.841
CRP (mg/L)	3.54 ± 3.05	3.12 ± 3.46	0.462
ESC Score	5.62 ± 2.16	5.86 ± 3.67	0.671

Data are presented as mean ± standard deviation (SD) for continuous variables and as number (percentage) for categorical variables. Significant coronary stenosis was defined as ≥50% luminal narrowing in any major epicardial coronary artery. Statistical comparisons: Student’s *t*-test for continuous variables, chi-square test for categorical variables. CAD = coronary artery disease; BMI = body mass index; LDL = low–density lipoprotein; HDL = high-density lipoprotein; CRP = C-reactive protein; ESC = European Society of Cardiology. *p*-values < 0.05 considered statistically significant.

**Table 2:** Univariate associations of significant coronary stenosis.

Variable	Odds Ratio	95% CI	*p*-Value
Age (per year)	1.007	0.970–1.051	0.687
Male sex	2.031	0.974–4.234	0.059
Female sex	0.492	0.236–1.026	0.059
BMI (per kg/m^2^)	1.003	0.929–1.030	0.841
Diabetes mellitus	2.364	1.046–5.339	0.039
Hypertension	0.671	0.337–1.316	0.237
Current smoker	1.513	0.768–3.167	0.274
History of CAD	1.285	0.681–2.696	0.482
Testosterone (per ng/mL)	1.197	1.064–1.346	0.003
LDL cholesterol (per mmol/L)	1.173	0.854–1.699	0.357
HDL cholesterol (per mmol/L)	0.759	0.571–1.008	0.059
Triglycerides (per mmol/L)	1.036	0.764–2.122	0.840
CRP (per mg/L)	1.043	0.938–1.281	0.461
ESC Score	0.975	0.867–1.096	0.670

Data are presented as odds ratios (ORs) with 95% confidence intervals (CIs) from univariate logistic regression analyses. All lipid ORs (LDL, HDL, triglycerides) are reported per 0.1 mmol/L increment for consistency. The outcome variable was binary: presence (1) or absence (0) of significant coronary stenosis, defined as ≥50% luminal narrowing in any major epicardial coronary artery.

**Table 3:** Testosterone levels stratified by maximum coronary stenosis severity.

Stenosis Category	*N*	Mean ± SD (ng/mL)	Median (IQR) (ng/mL)	*p*-Value
No stenosis (0%)	32	4.25 ± 3.55	4.25 (0.57–6.29)	(ref)
Mild (<50%)	25	5.62 ± 3.35	6.05 (3.76–7.57)	0.147
Moderate (50–69%)	20	6.12 ± 2.68	5.83 (4.36–7.76)	0.050
Severe (≥70%)	52	6.81 ± 2.84	7.29 (5.44–8.17)	<0.001

This table presents testosterone data as both mean ± SD and median with interquartile range across four stenosis severity categories. We defined these categories based on the most severe lesion found anywhere in the coronary tree. Abbreviations: SD = standard deviation; IQR = interquartile range (25th–75th percentiles); ref = reference group.

**Table 4:** Multivariate logistic regression analysis.

Variable	Coefficient	Std Error	OR	95% CI	*p*-Value
Testosterone (per SD = 3.22 ng/mL)	0.715	0.263	2.043	[1.221, 3.420]	0.007
Age (per SD = 9.71 years)	0.294	0.200	1.342	[0.907, 1.987]	0.141
Female sex	−0.105	0.495	0.900	[0.341, 2.374]	0.832
Diabetes mellitus	0.955	0.446	2.600	[1.085, 6.229]	0.032
LDL cholesterol (per SD = 1.01 mmol/L)	0.383	0.204	1.467	[0.985, 2.186]	0.060

OR = odds ratio per one-standard-deviation increase in variable. SD = Standard deviation: testosterone 3.22 ng/mL, age 9.7 years, LDL 1.01 mmol/L.

**Table 5:** Testosterone levels by number of diseased coronary vessels.

Number of Diseased Vessels	Overall (*n* = 129)	Testosterone (ng/mL)
0 (No disease)	n = 57	4.85 ± 3.50
1 (1-vessel)	n = 43	6.54 ± 2.87
2 (2-vessel)	n = 16	6.19 ± 3.15
3 (3-vessel)	n = 13	7.42 ± 1.99
Correlation (r)		r = 0.253, p = 0.004

**Table 6:** Published studies showing conflicting outcomes of testosterone-cardiovascular relationships.

Study	Design	Population	Main Finding	Proposed Mechanism
TRAVERSE (2023) [23]	Multi-center RCT, double-blind, placebo-controlled	Hypogonadal men (N = 5204)	Non-inferior to placebo for major CV events; testosterone safe in appropriate patients	Replacement of deficient hormone to physiological levels; no supraphysiological exposure
Corona et al. (2018) [12]	Meta-analysis	Men with low testosterone	Low testosterone predicts CV events and mortality	Testosterone deficiency as marker of poor health; reverse causation
Hudson et al. (2022) [7]	RCT	Men on testosterone treatment (various RCTs, individual patient data)	Increased adverse CV events and mortality during testosterone treatment	High-risk populations with pre-existing CVD; possible prothrombotic effects or unmasking of subclinical disease
EMAS (2013) [11]	Prospective cohort	Middle-aged/elderly European men	Low testosterone predicts incident CV events	Testosterone as marker of metabolic syndrome, inflammation, frailty
Trumble et al. (2023) [24]	Population-based	Indigenous population (stable CAD)	Higher testosterone in stable CAD patients vs. controls	Chronic compensatory upregulation; different from acute stress response
Current study (2024)	Cross-sectional, angiographic	Central European stable CAD (N = 129)	Paradoxical positive association: higher testosterone with more severe stenosis	Stable chronic CAD differs from acute; possible compensatory mechanism, ethnic differences

The contrasting outcomes reflect differences in study design (observational vs. interventional), population characteristics (acute vs. stable CAD, age, comorbidities), testosterone measurement (endogenous levels vs. replacement therapy), and clinical context. Abbreviations: RCT = randomized controlled trial; CV = cardiovascular; EMAS = European Male Ageing Study; CAD = coronary artery disease;.

## Data Availability

The original contributions presented in this study are included in the article. Further inquiries can be directed to the corresponding author.

## Abbreviations

The following abbreviations are used in this manuscript:

ACS	acute coronary syndrome
AR	androgen receptor
BMI	body mass index
CAD	coronary artery disease
CI	confidence interval
CRP	C-reactive protein
DHEA-S	dehydroepiandrosteron
DHT	dihydrotestosteron
EPV	events-per-variable ratio (EPV)
ESICD	East Slovak Institute for Cardiovascular Diseases
FSH	follicle-stimulating hormone
GDF-15	growth differentiation factor 15
HDL	high-density lipoprotein
HPG	hypothalamic-pituitary-gonadal
IQR	interquartile range
KSC MR	Košice Selective Coronarography Multiple Risk
LC-MS/MS	liquid chromatography-tandem mass spectrometry
LDL	low-density lipoprotein
LH	luteinizing hormone
NS	not significant
OR	odd ratio
PCOS	polycystic ovary syndrome
r	Pearson correlation coefficient
SHBG	sex hormone-binding globulin
TRT	testosterone replacement therapy

## References

[1] Roth G.A., Mensah G.A., Johnson C.O., Addolorato G., Ammirati E., Baddour L.M., Barengo N.C., Beaton A.Z., Benjamin E.J., Benziger C.P. (2020). Global Burden of Cardiovascular Diseases and Risk Factors, 1990–2019: Update From the GBD 2019 Study. J. Am. Coll. Cardiol..

[2] Brown J.C., Gerhardt T.E., Kwon E. (2023). Risk Factors for Coronary Artery Disease.

[3] Sud M., Chaudhry A., Qui F., Haldenby O., Godoy L.C., Austin P.C., Roifman I., Manuel D., Eurich D.T., Wijeysundera H.C. (2025). Sex Differences in Cardiovascular Health Status and Long-Term Outcomes in a Primary Prevention Cohort. JACC Adv..

[4] Kim S.H., Park J.J., Kim K.H., Yang H.J., Kim D.S., Lee C.H., Jeon Y.S., Shim S.R., Kim J.H. (2021). Efficacy of testosterone replacement therapy for treating metabolic disturbances in late-onset hypogonadism: A systematic review and meta-analysis. Int. Urol. Nephrol..

[5] Hackett G. (2019). Metabolic Effects of Testosterone Therapy in Men with Type 2 Diabetes and Metabolic Syndrome. Sex. Med. Rev..

[6] Jiang R., Wang Y. (2025). Association between Low Serum Testosterone Levels and All-cause Mortality in Patients with Cardiovascular Disease: A Study Based on the NHANES Database. Cardiovasc. Toxicol..

[7] Hudson J., Cruickshank M., Quinton R., Aucott L., Aceves-Martins M., Gillies K., Bhasin S., Snyder P.J., Ellenberg S.S., Grossmann M. (2022). Adverse cardiovascular events and mortality in men during testosterone treatment: An individual patient and aggregate data meta-analysis. Lancet Healthy Longev..

[8] Dobrzycki S., Serwatka W., Nadlewski S., Korecki J., Jackowski R., Paruk J., Ladny J.R., Hirnle T. (2003). An assessment of correlations between endogenous sex hormone levels and the extensiveness of coronary heart disease and the ejection fraction of the left ventricle in males. J. Med. Investig..

[9] Tang L., Chen M., Li J., Xu X., Pu X. (2024). Association of testosterone with myocardial infarction and severity of coronary artery disease among male patients. Int. J. Cardiol. Cardiovasc. Risk Prev..

[10] Kaur H., Werstuck G.H. (2021). The Effect of Testosterone on Cardiovascular Disease and Cardiovascular Risk Factors in Men: A Review of Clinical and Preclinical Data. CJC Open.

[11] Lee D.M., Pye S.R., Tajar A., O’Neill T.W., Finn J.D., Pendleton N., Silman A.J., Bartfai G., Casanueva F., Forti G. (2013). Cohort Profile: The European Male Ageing Study. Int. J. Epidemiol..

[12] Corona G., Rastrelli G., Di Pasquale G., Sforza A., Mannucci E., Maggi M. (2018). Testosterone and Cardiovascular Risk: Meta-Analysis of Interventional Studies. J. Sex. Med..

[13] Travison T.G., O’Donnell C.J., Bhasin S., Massaro J.M., Hoffmann U., Vasan R.S., D’Agostino R.B., Basaria S. (2016). Circulating Sex Steroids and Vascular Calcification in Community-Dwelling Men: The Framingham Heart Study. J. Clin. Endocrinol. Metab..

[14] Ackerman C.M., Lowe L.P., Lee H., Hayes M.G., Dyer A.R., Metzger B.E., Lowe W.L., Urbanek M., The Hapo Study Cooperative Research Group (2012). Ethnic variation in allele distribution of the androgen receptor (AR) (CAG)n repeat. J. Androl..

[15] Jones T.H., Kelly D.M. (2018). Randomized controlled trials—Mechanistic studies of testosterone and the cardiovascular system. Asian J. Androl..

[16] Shishkov S., Ivanova M., Hristov I., Tisheva S. (2022). Total testosterone levels in men with acute coronary syndrome. Scr. Sci. Med..

[17] Pesonen E., Pussinen P., Huhtaniemi I. (2016). Adaptation to acute coronary syndrome-induced stress with lowering of testosterone: A possible survival factor. Eur. J. Endocrinol..

[18] Pella Z., Pella D., Paralič J., Vanko J.I., Fedačko J. (2021). Analysis of Risk Factors in Patients with Subclinical Atherosclerosis and Increased Cardiovascular Risk Using Factor Analysis. Diagnostics.

[19] Vrints C., Andreotti F., Koskinas K.C., Rossello X., Adamo M., Ainslie J., Banning A.P., Budaj A., Buechel R.R., Chiariello G.A. (2024). 2024 ESC Guidelines for the management of chronic coronary syndromes. Eur. Heart J..

[20] Hostačná L., Mašlanková J., Pella D., Hubková B., Mareková M., Pella D. (2024). A Multi-Biomarker Approach to Increase the Accuracy of Diagnosis and Management of Coronary Artery Disease. J. Cardiovasc. Dev. Dis..

[21] SCORE2 Working Group and ESC Cardiovascular Risk Collaboration (2021). SCORE2 risk prediction algorithms: New models to estimate 10-year risk of cardiovascular disease in Europe. Eur. Heart J..

[22] Cooney M.T., Selmer R., Lindman A., Tverdal A., Menotti A., Thomsen T., Njølstad I., Veronesi G., De Bacquer D., De Backer G. (2016). Cardiovascular risk estimation in older persons: SCORE O.P. Eur. J. Prev. Cardiol..

[23] Lincoff A.M., Bhasin S., Flevaris P., Mitchell L.M., Basaria S., Boden W.E., Cunningham G.R., Granger C.B., Khera M., Thompson I.M. (2023). Cardiovascular Safety of Testosterone-Replacement Therapy. N. Engl. J. Med..

[24] Trumble B.C., Negrey J., Koebele S.V., Thompson M.E., Davison R., Kampp K., Gurven M., Kaplan H., Tsimane Health and Life History Project Teams (2023). Testosterone is positively associated with coronary artery calcium in a low cardiovascular disease risk population. Evol. Med. Public Health.

[25] Soleimany A., Kavandi H., Khalili N., Abbasi A., Ghaderi M., Karimi A. (2022). The Association Between Serum Testosterone Levels and Coronary Artery Disease in Men. Shiraz E—Med. J..

[26] Shi J., Bird R., Schmeling M.W., Hoofnagle A.N. (2021). Using mass spectrometry to overcome the longstanding inaccuracy of a commercially-available clinical testosterone immunoassay. J. Chromatogr. B Anal. Technol. Biomed. Life Sci..

[27] Meun C., Franco O.H., Dhana K., Jaspers L., Muka T., Louwers Y., Ikram M.A., Fauser B.C.J.M., Kavousi M., Laven J.S.E. (2018). High Androgens in Postmenopausal Women and the Risk for Atherosclerosis and Cardiovascular Disease: The Rotterdam Study. J. Clin. Endocrinol. Metab..

[28] Ikehata Y., Hachiya T., Kobayashi T., Ide H., Horie S. (2023). Body composition and testosterone in men: A Mendelian randomization study. Front. Endocrinol..

[29] Okobi O.E., Khoury P., De la Vega R.J., Figueroa R.S., Desai D., Mangiliman B.D.A., Colon O.L.V., Urruela-Barrios R.J., Abdussalam A.K., Diaz-Miret M. (2024). Impact of Weight Loss on Testosterone Levels: A Review of BMI and Testosterone. Cureus.

[30] Hirschberg A.L. (2024). Hyperandrogenism and Cardiometabolic Risk in Pre- and Postmenopausal Women—What Is the Evidence?. J. Clin. Endocrinol. Metab..

[31] de Medeiros S., Barbosa B., Lin Winck Yamamoto A., Winck Yamamoto M., Souto de Medeiros M., Soares Junior J., Baracat E.H. (2022). The interplay between androgens and adipocytes: The foundation of comorbidities of polycystic ovary syndrome. GREM Gynecol. Reprod. Endocrinol. Metab..

[32] Sood A., Hosseinpour A., Sood A., Avula S., Durrani J., Bhatia V., Gupta R. (2024). Cardiovascular Outcomes of Hypogonadal Men Receiving Testosterone Replacement Therapy: A Meta-analysis of Randomized Controlled Trials. Endocr. Pract..

[33] Budoff M.J., Ellenberg S.S., Lewis C.E., Mohler E.R., Wenger N.K., Bhasin S., Barrett-Connor E., Swerdloff R.S., Stephens-Shields A., Cauley J.A. (2017). Testosterone Treatment and Coronary Artery Plaque Volume in Older Men with Low Testosterone. JAMA.

[34] McCrohon J.A., Death A.K., Nakhla S., Jessup W., Handelsman D.J., Stanley K.K., Celermajer D.S. (2000). Androgen receptor expression is greater in macrophages from male than from female donors. A sex difference with implications for atherogenesis. Circulation.

[35] Lee J.H., Jung H.D., Choi J.D., Kang J.Y., Yoo T.K., Park Y.W. (2023). Non-linear association between testosterone and LDL concentrations in men. Andrology.

[36] Lakshman K.M., Kaplan B., Travison T.G., Basaria S., Knapp P.E., Singh A.B., LaValley M.P., Mazer N.A., Bhasin S. (2010). The effects of injected testosterone dose and age on the conversion of testosterone to estradiol and dihydrotestosterone in young and older men. J. Clin. Endocrinol. Metab..

[37] Bhasin S., Lincoff A.M., Basaria S., Bauer D.C., Boden W.E., Cunningham G.R., Davey D., Dubcenco E., Fukumoto S., Garcia M. (2022). Effects of long-term testosterone treatment on cardiovascular outcomes in men with hypogonadism: Rationale and design of the TRAVERSE study. Am. Heart J..

